# Projections of future forest degradation and CO_2_ emissions for the Brazilian Amazon

**DOI:** 10.1126/sciadv.abj3309

**Published:** 2022-06-15

**Authors:** Talita O. Assis, Ana Paula D. Aguiar, Celso von Randow, Carlos A. Nobre

**Affiliations:** 1 National Institute for Space Research (INPE), São José dos Campos, Brazil.; 2 Stockholm Resilience Centre, Stockholm, Sweden.; 3 Institute for Advanced Studies, University of São Paulo, São Paulo, Brazil.

## Abstract

In recent years, the area affected by forest degradation in the Brazilian Amazon has frequently been higher than deforestation. From August 2006 to July 2019, the degraded area totaled 194,058 km^2^, representing almost two times the 99,630 km^2^ deforested in the same period. The impacts of degradation include biodiversity loss and changes in the carbon stocks, affecting the CO_2_ balance and future climate changes. This paper aims to explore socioeconomic and environmental factors that influence forest degradation, project future scenarios, and assess the impact on the regional carbon balance, combining forest degradation and deforestation-related processes (clear-cut deforestation and secondary vegetation dynamics). We show that, while net CO_2_ emissions from 2020 to 2050 are 0.74 Gt CO_2_ in the Sustainable scenario, this value reached 22.63 Gt CO_2_ in the Fragmentation scenario, an increasingly plausible scenario given the recent trends in the region.

## Teaser

Land-use scenarios for the Brazilian Amazon show the net emissions varying from 0.74 to 22.63 Gt CO_2_ from 2020 to 2050.

## INTRODUCTION

Land changes are closely linked to sustainability and are critical drivers in the mediation between human and physical systems (*1*–*4*). Some of these processes consist of modification-land surface without conversion to a whole different land cover class. Forest degradation, which consists of partial forest loss due to anthropogenic actions or environmental changes, is an example of this type of process. It differs from clear-cut deforestation, in which the forest is substituted by pasture, for example.

In the Brazilian Amazon, the forest degradation process involves a combination of wood logging and fire (*5*–*8*), causing biodiversity loss (*9*, *10*), changes in forest structure (*11*), and carbon stocks (*12*–*15*), and other consequences. Degradation has been substantial in the Amazon in recent years, frequently affecting a larger area than deforestation (*16*). From August 2006 to July 2019, the degraded area totaled 194,058 km^2^, representing almost two times the 99,630 km^2^ deforested in the same period (*16*, *17*).

Land changes, such as degradation, affect local and global scales (*1*, *18*), motivating the analysis of their causes and consequences. These analyses can be supported by models that quantify the relationships between land changes and their drivers. Models help to organize knowledge and understand data relationships and their possible economic and environmental implications, in addition to enabling the evaluation of public policy options (*19*).

Models including interactions of land changes with climate, biodiversity, hydrological cycle, soil, or greenhouse gas (GHG) emissions are increasingly used to understand and represent human-nature interactions (*20*–*25*). Regarding CO_2_ emissions due to land-use changes, several models use different approaches. The bookkeeping model (*26*) represents the carbon flow from loss of initial biomass, where part of this biomass is burned, deposited as slash, or stored in products. This model is very useful to explore the impacts of different land-use processes, as it allows us to monitor and analyze post-forest disturbance dynamics. The model INPE-EM (*25*) presented an improvement of the bookkeeping model, representing it spatially. Last, Aguiar *et al.* (*27*) and Assis *et al*. (*28*) implemented CO_2_ emissions because of the forest degradation process in INPE-EM. In recent years, the need to reduce CO_2_ emissions to limit climate changes increases the demand for robust GHG emission estimates, especially in a sector with high emissions, such as land-use changes.

Combined with land change models, scenarios can help explore their impact under different socioeconomic and environmental conditions through plausible stories about the future. Some authors developed CO_2_ emission scenarios because of degradation in the Brazilian Amazon. Aguiar *et al.* (*27*) estimated forest degradation scenarios to 2050, however, without modeling the degradation driving factors. Longo *et al.* (*29*) explored the effects of the droughts on the forest to project scenarios to 2100. Fonseca *et al*. (*30*) and Le Page *et al*. (*31*) developed fire probability scenarios for 2100, combining land-use changes and climate scenarios.

This paper presents an innovative approach to creating forest degradation and CO_2_ emission scenarios, adapting and combining the land change model LuccME (*32*), the carbon emission bookkeeping model INPE-EM (*25*), and available deforestation scenarios. This approach allows us to explore socioeconomic and environmental driver factors that influence forest degradation spatial distribution, project future scenarios of degradation, and estimate CO_2_ emissions for each scenario for the Brazilian Amazon.

## RESULTS

The experiments performed to achieve the objective of this work generated partial results that are important to be analyzed. Therefore, this section presents the statistical analysis that was input to the LuccME, the results from the degradation model from 2006 to 2019 and its validation, the degradation scenarios, and, finally, the scenarios for 2050 and CO_2_ emissions.

### Forest degradation driver factors

The statistical analysis performed from 2007 to 2011 to support the LuccME model potential component is important to understand the spatial distribution of degradation in the Amazon. The exploratory analysis of the data pointed the need to build different models for the dry and nondry years because of the differences in the degradation process between the two periods, discussed in (*33*). We developed three Spatial Lag regression models aiming to understand the driving factors of forest degradation: a model for the forest degradation in years with extreme drought, a model for the forest degradation in nondrought years, and a model for the nondegraded forest, to verify factors that help prevent this type of event. Table 1 describes these models and their *R*^2^ and Akaike Information Criterion (*34*).

**Table 1.** Spatial lag regressions.

**Nondegraded forest**			**Degradation in normal precipitation years**			**Degradation in extreme drought years**		
***R*^2^: 0.86**			***R*^2^: 0.59**			***R*^2^: 0.46**		
**Akaike (AIC): −8,031**			**Akaike (AIC): −16,099.5**			**Akaike (AIC): −27,777.8**		

**Variable**	**Coefficient**	**Prob.**	**Variable**	**Coefficient**	**Prob.**	**Variable**	**Coefficient**	**Prob.**
Spatial coefficient	0.90	0.000	Spatial coefficient	0.824	0.000	Spatial coefficient	0.773	0.000
Constant	−0.111	0.000	Constant	0.066	0.000	Constant	0.000	0.102
Conservation units	0.052	0.000	Historical deforestation	0.024	0.000	Water deficit anomaly	−2.834 × 10^−5^	0.000
Indigenous territories	0.057	0.000	Connection to markets	−0.004	0.000	Recent deforestation	0.837	0.000
Roads	0.006	0.000						
Urban centers	0.008	0.000						

Degradation was best explained by Historical Deforestation and Connection to Markets in nondrought years and Water Deficit Anomaly, and Recent Deforestation in drought years. The variables that best described the permanence of the nondegraded forest were the percentage of Conservation Units, Indigenous Territories, and distance to Roads and Urban Centers. The spatial coefficient, which measures the spatial correlation of each dependent variable, was substantial and higher than 0.75 in all models, meaning that degradation is also a spatially concentrated process, as deforestation (*35*). We obtained 0.59, 0.46, and 0.86 of *R*^2^ score to the degradation in nondrought years, degradation in extreme drought years, and nondegraded forest, respectively.

### Land change model validation

**Fig. 1.**
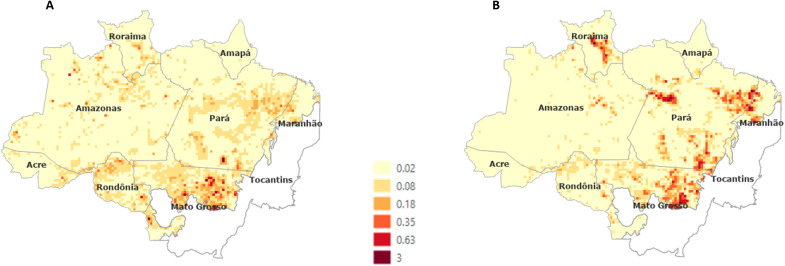
Percentage of degradation in 25 km × 25 km cells from 2012 to 2019. (**A**) Simulated by LuccME. (**B**) Real data.

We ran the LuccME model from 2012 to 2019 using the regression models presented above and compared the simulated degradation maps with the real degradation data to verify the adjustment. Figure 1 shows maps of the percentage of degradation in 25 km × 25 km cells from 2012 to 2019 simulated by model (Fig. 1A) and inferred by DEGRAD and DETER degradation data (Fig. 1B). The model fit reached 66.6% when comparing the patterns of both maps.

In general, the model captured all the leading hot spots of degradation in the period. The model represented the degradation patterns in northern Mato Grosso state, a region with most of the degradation observed in the period. However, the model overestimated degradation in Rondônia and underestimated it in Pará and Roraima states, especially in southeastern Pará state. On the basis of these models, we proceeded to explore future scenarios.

### Forest degradation and CO_2_ emission scenarios

We built two degradation scenarios, Sustainable, with improvements in the socioeconomic, institutional, and environmental dimensions, and Fragmentation, with the weakening of the socioenvironmental dimension and chaotic urbanization, combining the premises of Deforestation Scenarios developed by (*27*) with the Fire Probability Scenarios presented by (*30*).

The deforestation Sustainable scenario (*27*) considered that political and institutional conditions would favor reducing deforestation by 2020. This scenario also considers the regeneration of all illegally deforested areas and assumes that secondary vegetation will increase from 22 to 35% from 2015 to 2030 and will no longer be periodically removed. Based on the (*30*) estimates in “RCP4.5 and Sustainable scenario,” we calculated forest degradation rates considering the increase of 90.9% in the forest degradation until 2100.

The deforestation Fragmentation scenario (*27*) assumed a return of high deforestation rates, like those before 2004. In this scenario, the National Forest Code is not respected. Secondary vegetation follows its current dynamics, with a high rate of deforested land abandonment and a short cutting cycle in consolidated areas. We calculated the forest degradation rates considering the increase of 21% in the forest degradation until 2100, estimated on the (*30*) estimates in “RCP4.5 and Fragmentation scenario.”

**Fig. 2.**
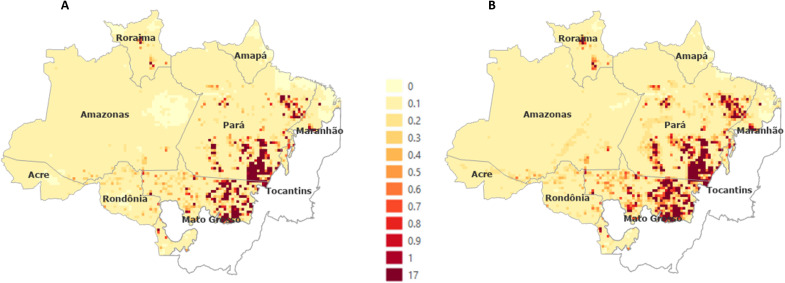
Percentage of degradation in 25 km × 25 km cells from 2020 to 2050. (**A**) Sustainable scenario. (**B**) Fragmentation scenario.

Figure 2 shows the maps containing the total percentage of degradation in 25 km × 25 km cells, which occurred from 2020 to 2050 for Sustainable and Fragmentation degradation scenarios. As the maps represent the sum of the degradation that occurred in each cell within the period of the scenarios, values greater than 1 (one) may occur, which indicates that this cell has suffered recurrent degradation.

We note repeated degradation events in northern Mato Grosso state and the southeastern and northeastern Pará state, being the most affected areas by degradation in both scenarios. We emphasize that these areas also should be the most affected by deforestation by 2050, according to (*27*) scenarios. In other words, in these regions, an intensification of the patterns already observed today is expected, with a large part of the forest exposed to deforestation or forest degradation.

Almost 100% of forest cells are exposed to some level of degradation by 2050. At the end of the simulation, most grid cells had up to 10% of forest degradation. However, in the Sustainable scenario, it is still possible to observe regions of intact forest, especially in eastern Amazonas state.

We combined the Sustainable and Fragmentation degradation scenarios with the Sustainable and Fragmentation deforestation scenarios provided by (*27*) to perform integrated CO_2_ emission estimates. Using the INPE-EM model (*27*, *28*), which associates land-use and biomass change maps to calculate CO_2_ emissions, we projected the carbon balance in Sustainable and Fragmentation scenarios. Within the scenarios period (from 2020 to 2050), CO_2_ net emissions totaled 1.3 Gt CO_2_ in the Sustainable scenario and 24.07 Gt CO_2_ in the Fragmentation scenario, respectively, considering the emissions from forest degradation and deforestation, gains from degraded forest recovery, and secondary vegetation growth and emission from secondary vegetation loss. The gross emission due to forest degradation projected from 2020 to 2050 was 6.9 and 10.2 Gt CO_2_ in Sustainable and Fragmentation scenarios, respectively, representing 46.7 and 25.4% of the 14.77 and 40.18 Gt CO_2_ gross emissions in Sustainable and Fragmentation scenarios. Table 2 summarizes these results.

**Table 2.** CO_2_ balance simulated in the scenarios.

**CO_2_ (Gt CO_2_)**	**Period**	**Sustainable scenario**	**Fragmentation scenario**
Net emissions	1960–2019	37.23	36.99
	2020–2050	1.31	24.07
Degradation emissions	1960–2019	4.65	4.50
	2020–2050	6.9	10.22
Total emissions	1960–2019	44.48	43.85
	2020–2050	14.77	40.18
Degraded forest regeneration	1960–2019	−2.18	−1.97
	2020–2050	−6.74	−8.55

## DISCUSSION

This paper presented an innovative approach to creating degradation and CO_2_ emission scenarios, adapting and combining the LuccME land change model and the INPE-EM CO_2_ emission model. We organized the discussion into three parts. We first discussed the land change modeling implications of our results, then the spatial drivers of change, and, finally, the scenario results.

### Modeling different behavior in drought and nondrought years

Our results demonstrated that the degradation assumed two distinct behaviors over the analysis period, one for years of extreme droughts and another for nondrought years. For this reason, we modified the LuccME framework to use two separate regressions for degradation. For this, we include a decision rule that makes the model alternate between both regressions throughout the simulation.

This approach enables exploration of the socioeconomic and environmental factors that influence forest degradation spatial distribution and project scenarios of degradation and CO_2_ emissions of Brazilian Amazon. The adaptations made in the LuccME model to represent forest degradation can be used in other processes with similar characteristics.

### Drivers of degradation

Among the various socioeconomic and environmental factors analyzed in this work, historical deforestation and the connection to markets (*25*, *35*) better explained the spatial distribution of degradation in nondrought years, reinforcing the understanding of the influence of historical deforestation on degradation. The Fragmentation scenario caused by clear-cut deforestation exposes the forest along the edges (*36*) because of environmental or (*8*, *37*) anthropogenic factors (*38*). Environmental drivers include the increase of flammability, wind speeds, and insolation rates. Anthropogenic drivers include facilitating the flow of wood and the management of areas deforested with fire, which can spread into the forest.

The market connects local activities, such as crops, for example, to regional and global processes (*39*, *40*). Aguiar *et al.* (*35*) point out the connections to markets as an important factor in capturing the spatial patterns of the new frontiers in Brazil. The variable “connection to markets” used in this paper was constructed using the Generalized Proximity Metrics (GPM) to calculate the relative distance from each cell of the cellular space to São Paulo or Recife cities throughout the roads. It is essential to assess how connected to the main consumption markets each cell is. Our results also pointed out the importance of this driver to the degradation process. The “Connectivity to markets” variable was created calculating the distance from each cell to Sao Paulo or Brazilian Northeast considering the paved and unpaved roads. By considering the distance between two points weighted by highways, it combines consumer centers and roads to compose a connectivity indicator.

In years of extreme drought, the analyses pointed to water deficit anomaly and recent deforestation as the substantial drivers of forest degradation. Several authors have pointed out the importance of the relationship between water deficit in years of extreme drought (*41*–*43*). In these years, the proximity to recent deforestation (which occurred in the same year) gains space because of the escape of fires resulting from the cleaning of deforested areas. As the areas are drier, the fire spreads more easily, entering the forest regions.

### Land-use/cover change and CO_2_ emission scenarios

This paper developed an innovative approach to creating future scenarios of forest degradation and corresponding CO_2_ emissions, adapting the land change modeling framework LuccME and combining it to INPE-EM emission models. This approach allowed us to explore socioeconomic and environmental factors that influence the spatial distribution of forest degradation and project scenarios of degradation and CO_2_ emissions to the Brazilian Amazon. Merging degradation scenarios with deforestation scenarios developed by (*27*) allowed an integrated CO_2_ emission estimates.

This work presented two scenarios of emissions from forest degradation, considering two land-use scenarios, based on the Sustainable and Fragmentation scenarios of (*27*), combined with “Fragmentation + RCP4.5” and “Sustainable + RCP4.5” scenarios of (*30*), which had previously simulated forest degradation scenarios from land-use changes and climate change. Le Page *et al.* (*31*) also developed degradation scenarios based on land use and climate change for the Amazon. Still, both scenarios do not include the emissions resulting from this process. Aguiar *et al.* (*27*) modeled the emissions resulting from forest degradation, but the degradation was not spatially modeled in those scenarios. Our results show a wide variation in the carbon balance between the two scenarios and bring gains in understanding how changes in deforestation/secondary vegetation/degradation patterns can affect CO_2_ emissions in the Amazon.

Given the importance of deforestation as a driver, deforestation resulting from these scenarios substantially affects degradation results. The Sustainable Scenario is quite close to the Brazilian nationally determined contribution (NDC), which pledge on zero illegal deforestation by 2030. However, the resumption of growth in deforestation recorded by the PRODES system in recent years (e.g., 2019, 2020, and 2021) takes Amazon reality away from the Sustainable scenario, bringing us closer to the Fragmentation scenario.

To explore the impact of different land-use change scenarios on emissions from degradation, we chose to work only with RCP4.5. However, as an improvement for future work, we suggest the use of different climate scenarios.

## MATERIALS AND METHODS

### Experimental design

**Fig. 3.**
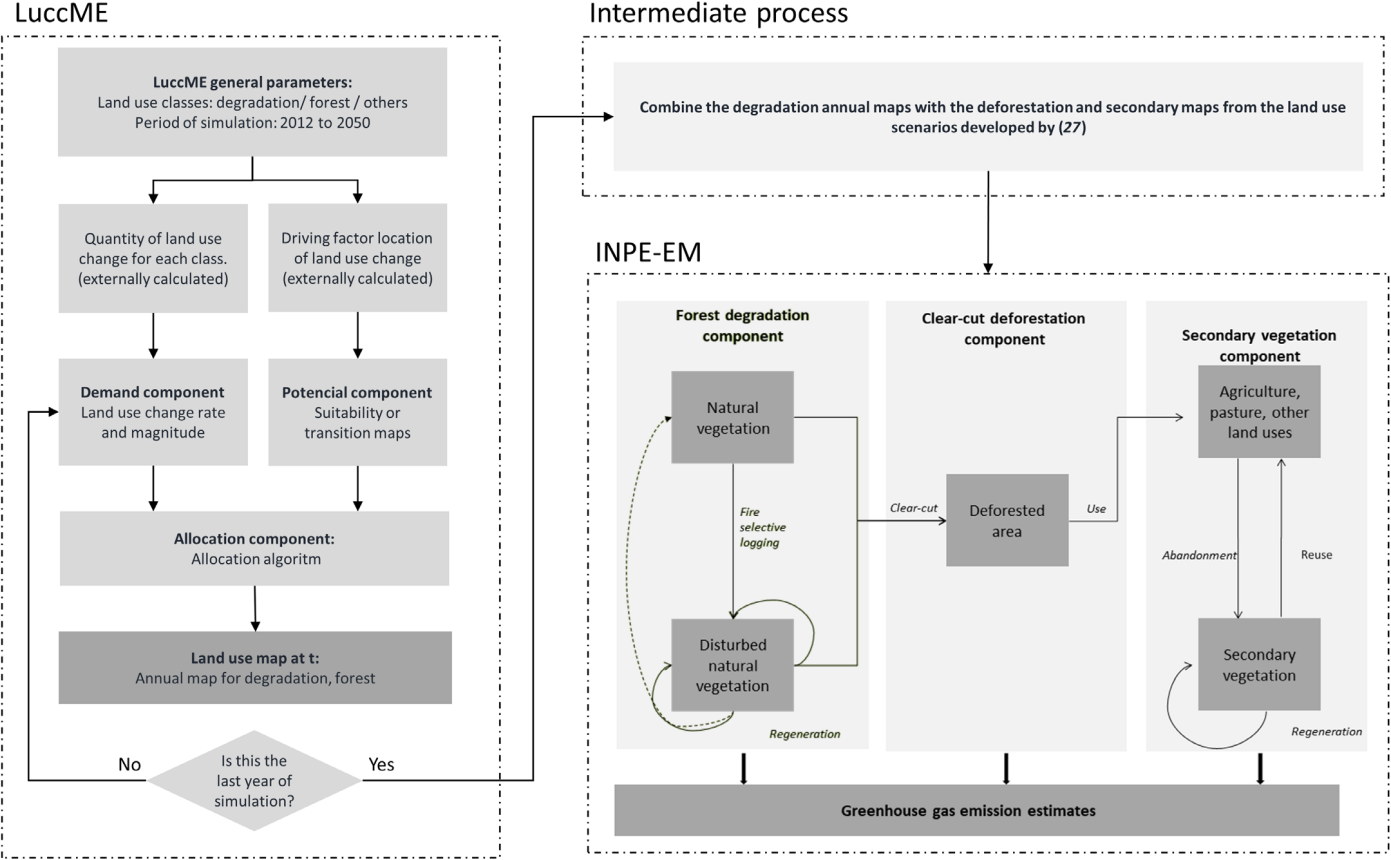
Experimental design. LuccME modeling framework generates the annual land-use maps, and INPE-EM represents the degraded forest dynamics and calculates CO_2_ emissions derived from this process. Adapted from (*32*) and (*28*).

To discuss scenarios of CO_2_ emissions from forest degradation in the Amazon in the period 2020–2050, we combined a spatially explicit land-use modeling approach with the CO_2_ emission model INPE-EM (*25*, *28*). We used the LuccME land-use modeling framework (*32*) to generate the annual degradation maps until 2050, combined this maps with clear-cut deforestation and secondary vegetation scenarios developed by (*27*), and calculated CO_2_ emissions, considering the clear-cut deforestation and forest degradation processes, using INPE-EM, as illustrated in Fig. 3.

### Modeling tools

#### LuccME

LuccME is an open-source framework for the development of dynamic spatially explicit land-use and cover change models. This type of model can describe the evolution of land use and cover spatial patterns over time, quantifying its drivers (*44*) and spatially allocating the demand for change according to the potential of each cell. In general, we can divide these models into three components: demand, potential, and allocation. The Demand Component defines the change that the model will allocate at each time step (*45*, *46*). Demand can be calculated from historical trends, assumptions arising from scenario construction, or economic models. The Potential Component is based on explanatory variables, mainly related by empirical methods, to calculate the suitable changes for each cell, defined by the demand component. The Allocation component is composed of computational mechanisms that establish competition through decision rules to allocate demand according to the potential of each cell at each model time step. LuccME separates these components responsible for calculating demand, potential, and allocation mechanisms and implements different components, according to the concepts of the various models found in the literature. We used the LuccME components derived from the CLUE model for continuous land-use variables (*47*) to generate annual degradation maps. In the “LuccME parametrization” section, we detailed Demand, Potential, and Allocation components and explain how we parametrized each of them at this work.

#### INPE-EM

INPE-EM is a carbon emission model (*25*) based on the bookkeeping model proposed by (*26*) and aims to generate annual estimates of emissions of GHG by the land cover change in a spatially explicit way. It is composed of three components: (i) clear-cut deforestation, (iii) secondary vegetation, and (iii) forest degradation, which permits the representation of emission processes in an integrated way (*27*). We used the degradation maps generated in the LuccME, combined with deforestation and secondary vegetation maps developed by (*27*) to obtain the complete estimates.

Here, we used the INPE-EM degradation component implemented by (*28*). This component improves the representation of the biomass changes following a degradation event and allows the use of different growth curves to represent the regeneration of the aboveground biomass (AGB).

We considered the second-order emission estimates provided by INPE-EM, which represent the gradual process of liberation and carbon absorption through several years after land change events and therefore carry the influence of lagged emissions because of historical processes in previous years.

#### Study area and input data

Our study area is the Brazilian portion of the Amazon Biome, monitored by the PRODES system (*16*), which corresponds to approximately 4,000,000 km^2^. We used INPE monitoring systems, PRODES, DEGRAD, and DETER (*16*, *17*), as our land cover sources. The PRODES system (*16*) identifies clear-cut deforestation using satellite monitoring in the Brazilian Legal Amazon since 1988 and is considered the official data for Amazon deforestation by the Brazilian government. Once an area is identified as deforested, it is not monitored in the following years, even if crops or livestock farming in these areas is eventually abandoned, and there is a regeneration of the forest vegetation.

The DEGRAD system identifies forests exposed to fires and disordered selective exploitation in the areas monitored by PRODES. Degradation, unlike deforestation, is mapped in the year in which it occurred but does not remain represented in the following year. That is, while deforestation in 2007 corresponds to accumulated deforestation up to that year, degradation in 2007 corresponds to degradation that occurred exclusively in that year, without considering what happened in previous years. As degradation is not a complete land cover modification, the same forest area can repeatedly suffer degradation and remain considered a forest. PRODES and DEGRAD generate annual products based on Remote Sensing imagery acquired from August of the prior year to July.

DETER B maps deforestation and other changes in forest cover and was used to complete the degradation temporal series because the DEGRAD system was discontinued in 2016 and replaced by DETER. To be compatible with the definitions of areas mapped by DEGRAD, we used the DETER B “Degradation” and “Burnt scar” classes (*17*). We also considered the period from August 1st of the previous year to July 31st for each year of analysis, like PRODES and DEGRAD.

To represent the degradation through a spatially explicit model, we organized a set of variables related to forest degradation based on the literature. These variables were integrated into a cellular space of 25 km × 25 km to make compatible information from different sources and formats. The cellular space (*48*) is a matrix structure where each cell is associated with several types of attributes, allowing to associate the vector and raster data in a single data layer within a Geographic Information System. Table 3 shows the dataset, its source, and how it was stored in the cellular space.

**Table 3.** Variables and data sources.

**Variable**	**Representation in the** **cellular space**	**Source**
Degradation	Percentage of the cell degraded at year *t*	DEGRAD and DETER (*16*, *17*)
Historical deforestation	Percentage of the cell deforested until the previous year *t* − 1	PRODES (*16*)
Recent deforestation	Percentage of the cell deforested at year *t*	PRODES (*16*)
Water deficit anomaly	Water deficit anomaly at year *t*	(*64*, *65*)
Slope	Slope average in the cell	(*66*)
Fertility	Fertility average in the cell	(*67*)
Roads	Euclidean distance to the nearest road	(*68*)
Connection to markets	Distance to Sao Paulo or Brazilian Northeast considering the paved and unpaved roads	(*27*, *35*, *68*)
Railways	Euclidean distance to the nearest railways	(*68*)
Hydroways	Euclidean distance to the nearest hydroways	(*69*)
Distance to the wood poles	Euclidean distance to the nearest wood poles	(*70*)
Mining (concession)	Euclidean distance to the nearest mining site	(*71*)
Small-scale/alluvial (“Garimpo”) mining (concession)	Euclidean distance to the nearest small-scale mining site	(*71*)
Urban centers	Euclidean distance to nearest urban centers with more than 10,000 inhabitants	(*72*)
Rural settlements	Percentage of the cell coverage by rural settlements	(*73*)
Indigenous territories	Percentage of the cell coverage by conservation units	(*74*, *75*)
Conservation units	Percentage of the cell coverage by indigenous territories	(*76*)
Hydroelectric plants under construction or operation	Euclidean distance to hydroelectric plants under construction or operation	(*77*)

### Statistical analysis

#### LuccME parametrization

For the LuccME parametrization, we divided the study area into three different land cover classes: forest, degradation, and others, which includes nonforest areas and areas classified as “deforested” by the PRODES system (*16*). The “others” class is not simulated by the model but is considered a “mask” applied over the study area, updated every year.

The land-use classes were represented in the cellular space by the percentage of each one contained in each cell. The period considered in the model is from 2007 to 2050, distributed as follows: (i) 2007–2011 (used to calculate the potential of each cell), (ii) 2012–2019 (model calibration and validation), and (iii) 2020–2050 (model simulations). The following sections present the parametrization for LuccME Demand, Potential, and Allocation components.

#### Demand

We used LuccME Pre Computed Values Component, in which we externally calculate the demand and inform the model the expected area for each land cover each year. The degradation demand corresponds to the annual degradation area indicated by DEGRAD (until 2016) and DETER (after 2016). The forest area for each year is the forest area (considered in the respective PRODES year) minus the degraded area that year.

#### Potential (statistical analysis)

To spatially distribute the demand for each land cover class in the model domain, LuccME also calculates the potential of occurrence of a given land cover class (*25*). In this work, we used the LuccME potential component based on spatial regression (*49*), where the dependent variable is influenced by its occurrence in the neighborhood because changes in land use/cover in an area tend to spread through neighboring regions. This component allows us to dynamically update the potential for changes at each time step, considering the temporal changes in the spatial drivers and the occurrence of degradation in previous years. Table 1 presents the list of potential spatial drivers we considered in our analysis.

We weighted the degradation in each cell by its respective forest area to avoid contamination by deforested and nonforest data for the construction of the spatial regression model. Therefore, we excluded from statistical analysis cells whose forest percentage was equal to zero. We also performed a Spearman correlation analysis (*50*) between the variables in our dataset to prevent the use of factors with a correlation coefficient above 0.6 in the same regression.

We performed the statistical analysis for degradation considering the period from 2007 to 2011 and adjusted the explanatory variables to this date. On the basis of the literature (*42*, *51*), we explore the degradation drivers considering two distinct periods: (i) from 2007 to 2010 (years of degradation not affected by drought) and (ii) 2011 (year of degradation affected by extreme drought year). The 2011 year represents the influence of the extreme drought of 2010 (*52*). We emphasize that, according to DEGRAD and PRODES methodology, the degradation mapped each year goes from August of the previous year to July of that year. Thus, 2011 degradation, for example, refers to the period from August 2010 to July 2011.

During the exploratory analysis phase, we observed that the spatial distribution of degradation was markedly different in “nondrought” and “drought” years. On the basis of this conclusion, we explored separate regressions. We adapted LuccME to alternate between both regressions during model execution using an attribute that indicates whether the year was a nondrought or drought impacted year. According to this rule, we set the “2010 average water deficit anomaly” as a threshold to classify all years. Years with an average value lower than the 2010 average water deficit anomaly were considered drought years, while the others were considered nondrought years. The model fit was assessed using the multiple determination coefficient values (*R*^2^) and the Akaike Information Criterion (*34*) that show the fit of the model (*49*).

#### Allocation

Once we have defined the model demand and potential parameters, we applied the LuccME allocation component (AllocationCClueLike) based on CLUE (*47*) to allocate the land cover classes annually for the period from 2012 to 2019. The model was configured so that for each year, both the forest and degradation classes may increase or decrease the area occupied within the cells, reflecting the behavior observed in the real data. The others class was adjusted annually to incorporate the deforestation that occurred over the years.

Once we defined all the parameters, we ran the model for the period from 2012 to 2019 to assess whether we were able to capture the behavior observed in the degradation data. To evaluate the results, we used the validation method of adjustment of multiple spatial resolutions (*53*), which establishes the similarity between the simulated map and the real map in different resolutions. This approach allowed us to evaluate both location errors in the model resolution itself and spatial pattern errors, degrading the resolution of the maps.

#### INPE-EM parametrization

INPE-EM combines land cover and biomass change maps to calculate CO_2_ emissions. We used the annual maps of degradation resulting from the simulation with LuccME and the deforestation and secondary vegetation scenarios developed by (*27*) to estimate CO_2_ emissions from 2016. Estimates of CO_2_ emissions were based on (*28*) parameters described in Table 4.

**Table 4.** Parameters settings for the INPE-EM degradation component. AGB, aboveground biomass; BGB, belowground biomass.

**Parameter**	**Description**	**Nonspatial**	**Spatial**
Biomass	Average biomass in a cell unit	233 ton ha^−1^	Brazilian Third National GHG Inventory (*54*)
Degradation	Percentage of cell unit identified as degraded that year by fire/logging events	155,872 ha (*55*)	DEGRAD (*16*)
AGB loss	Percentage of AGB lost as a result of the event	54.2% (*14*)	54.2% (*14*)
BGB loss	Percentage of BGB lost as result of the event	0	0
Deadwood loss	Percentage of deadwood lost as a result of the event	46.9% (*57*)	46.9% (*57*)
Litter loss	Percentage of litter lost as result of the event	46.9% (*57*)	46.9% (*57*)
Growth curves	Rates of regeneration of the AGB along the years	Based on (*14*) relationship between intact and 1× burned forests	Based on (*14*) relationship between intact and 1× burned forests

We used INPE-EM nonspatial mode from 1960 to 2006 and spatial mode from 2007 to 2019. To account for lagged emissions and historical disturbances in the Amazon forest, we used historical nonspatial data, based on the literature for the 1960–2006 period, following the approach adopted in (*25*).

We adopted the biomass data from the Brazilian Third National GHG Inventory (*54*). The average AGB used for the historical nonspatial period was 233 ton/ha, corresponding to the average of AGB in the areas that were degraded in the past.

We used the DEGRAD (until 2016) and DETER (after 2016) systems to provide degradation data for the INPE-EM spatial mode. For the nonspatial mode, we used the results of (*55*), which assessed an area of 1,714,600 ha degraded forests in the Brazilian Amazon between 1988 and 1998. We considered a homogeneous annual average value (155,872 ha) for the period from 1988 to 2006. No degradation was considered before this period (1960–1987), following (*27*).

We used the relationship between intact and degraded forests over the years described by (*14*) to represent the biomass loss and recovery in a degraded area. Their results presented the biomass changes following conventional logging and fire pathways. We adopted the “1 time burned (average),” and based on it, we defined the AGB loss and generated the AGB regeneration curve. The recovery rates obtained from this curve are comparable with others presented in the literature (*43*, *56*). We also used the results of (*57*) to define litter and dead wood loss.

#### Scenarios

To generate the degradation scenarios, we rely on the deforestation and secondary vegetation scenarios developed by (*27*), based on the Story and Simulation (SAS) approach (*58*), which combines qualitative and quantitative elements. These scenarios were produced with the participation of stakeholders in structured workshops (*59*) to discuss desired and undesirable visions of the future related to natural resources, social development, economic activities, infrastructure, technology, and the political and institutional context. From these visions of the future, trajectories were constructed to reach each of them, thus defining the scenarios: (i) Sustainable, with improvements in the socioeconomic, institutional, and environmental dimensions; (ii) Fragmentation with the weakening of the socioenvironmental dimension and chaotic urbanization; and (iii) “middle of the road.” These scenarios are reasonably aligned with the Intergovernmental Panel on Climate Change (IPCC) Shared Socioeconomic Pathways (SSP) 1, 3, and 2, respectively (*60*, *61*). In this work, we considered the “Sustainable” and Fragmentation scenarios and their premises to generate the degradation scenarios and combine them with the scenarios produced by (*27*).

We selected four elements for the qualitative narratives from (*27*) to be represented in the quantitative models: (i) environmental law enforcement, (ii) future clear-cut deforestation, (iii) secondary vegetation dynamics in abandoned areas after clear-cut deforestation, and (iv) changes in the substantial spatiotemporal deforestation drivers: conservation units and roads (*35*). We adopted all these premises described in (*27*) in the development of forest degradation scenarios. The Sustainable scenario considered that political and institutional conditions would favor reducing deforestation by 2020, reaching an average of 1000 km^2^/year from 2025 onward. This scenario also considers the regeneration of all illegally deforested areas and assumes that the secondary vegetation will increase from 22 to 35% from 2015 to 2030 and will no longer be periodically removed. The Fragmentation scenario took a return of high deforestation rates, like those before 2004, of 15,000 km^2^/year. In this scenario, the National Forest Code is not respected. Secondary vegetation follows its current dynamics, with a high rate of deforested land abandonment and a short cutting cycle in consolidated areas.

To define forest degradation rates in each of the scenarios, we applied the results of (*30*). They projected forest degradation until 2100 using (*27*) land-use scenarios and climate scenarios based on Representative Concentration Pathways RCP4.5 and RCP8.5 (*62*, *63*). To explore variations in socioeconomic assumptions and deforestation scenarios, we adopted a single scenario RCP4.5, which considers the stabilization of the radiative forcing at 4.5 W m^−1^ in the year 2100. As we adopted two opposing land-use scenarios, we decided to use an intermediate climate scenario for both.

We calculated the annual amount of degradation in each scenario by applying the projected growth rate for each scenario to the yearly reference value, given by the average degradation between 2007 and 2019. We, therefore, adopted (*30*) degradation growth projections, which combined (i) RCP4.5 and the Sustainable scenario and (ii) RCP4.5 and the Fragmentation scenario.

Last, we used the annual degradation maps generated for each scenario up to 2050 to estimate CO_2_ emissions resulting from this process. For this, we used INPE-EM (*25*) with the parameters of deforestation and secondary vegetation adopted in (*27*) scenarios and the parameters of degradation described in the “INPE-EM parametrization” section.
